# International health regulations and pre-travel health practices of international travelers at Nigerian airport: a cross-sectional study

**DOI:** 10.1186/s40794-023-00207-8

**Published:** 2023-12-05

**Authors:** Oluwatosin Samson Jegede, Grace Ijitade, Oyedoyin Aanu Fatoye, Timilehin Mercy Jegede, Nicholas Aderinto, Oluwafunmilayo Adenike Oguntoye, Oluwatosin Oluwagbenga Oguntoye, Oluwatosin Ruth Ilori, Olugbemiga Lanre Abodunrin, Adenike Iyanuoluwa Olugbenga-Bello, James Bamidele, Dauda Bayo Parakoyi

**Affiliations:** 1 Herbert Wertheim School of Public Health and Human Longevity Science, University of California, San Diego, CA USA; 2 Department of Biostatistics and Epidemiology, College of Public Health, Johnson City, East Tennessee State USA; 3 Department of Community Medicine, LAUTECH Teaching Hospital, Ogbomoso, Nigeria; 4 Department of Anesthesia and Intensive Care Unit, Obafemi Awolowo University Teaching Hospitals Complex, Ile-Ife, Nigeria; 5 Department of Medicine and Surgery, Ladoke Akintola University of Technology, Ogbomoso, Nigeria; 6 Department of Medicine, Afe Babalola University, Ado-Ekiti, Nigeria; 7 Department of Community Medicine, College of Health Sciences, Ladoke Akintola, University of Technology, Ogbomoso, Nigeria; 8 Department of Community Medicine, Ekiti State University, Ado Ekiti, Nigeria

**Keywords:** Airports, Vaccinations, International Health Regulation, Travelers, Pre-travel health practices

## Abstract

**Background:**

International Health Regulations (IHR) were developed by the World Health Organization (WHO) to curb the trans-border spread of epidemics. To our knowledge, no airport-based studies have assessed travelers’ health practices against a combination of diseases subject to IHR 2005. Therefore, we aimed to generate and describe the baseline travelers’ pre-travel health practices towards Cholera, Yellow Fever (YF), and Plague at Murtala Muhammed International Airport (MMIA) in Nigeria.

**Methods:**

A cross-sectional study was employed to collect data from 486 international travelers using a multistage sampling technique. Pre-travel health practices (a combination of pre-travel consultation, pre-travel vaccination, and preventive measures against insect bites) were assessed using an interviewer-administered questionnaire. Logistic regression models were used to estimates the association between selected variables and pre-travel health practices. Statistical significance level was set at 5%.

**Results:**

A total of 479 complete questionnaires were analyzed. The median age of respondents was 34.0 years Interquartile range (IQR) = 28.0, 44.0). Of the total respondents, 311 (64.3%) were aware of pre-travel health consultation and sources of information, amongst others, including friends/relatives in 180 (37.6%) travelers, social media/internet in 155 (32.4%) travelers, and health professionals in 102 (21.3%) travelers. Two hundred and seventy-one (56.6%) had pre-travel consultation, 156 (32.6%) had YF vaccination, and 226 (47.2%) were prepared to use preventive measures against insect bites. Only 10.6% had good pre-travel practices against the diseases subject to 2 International Health Regulations (IHR). Travelers with bachelor/college degrees, when compared to those with secondary/high education, had 2.91 times higher odds of having good practices when adjusting for other factors (95% C.I: 1.10, 7.70; p < 0.03). Also, those traveling to destinations endemic for YF infection, when compared to those who are not traveling to endemic countries/areas, had 48% lower odds of having good practices after adjusting for other factors (95% C.I: 1.41, 7.77; p < 0.01).

**Conclusions:**

Our study revealed a low prevalence of good pre-travel health practices among participants. Educational level and endemicity of YF at the destination were predictors of pre-travel health practices. Introducing topics on travelers’ health into schools’ curriculums may have a ripple positive effect on health practices among international travelers. Also, there is a need for public enlightenment programs on pre-travel health practices using social media platforms.

**Supplementary Information:**

The online version contains supplementary material available at 10.1186/s40794-023-00207-8.

## Introduction

International Health Regulation is a set of guidelines developed by the World Health Organization (WHO) to prevent the transnational spread of deadly epidemics. Compared to the IHR released in 1969, the latest IHR 2005 is better suited to current trends in the epidemiology of infectious diseases and new and re-emerging health risks [1]. The IHR 2005 includes diseases like Cholera, Pneumonic Plague, Yellow Fever (YF), Viral hemorrhagic fevers like Ebola, Lassa, and Marburg, and other potential public health concerns such as those with an unknown cause or source [2].

International travelers are at risk of contracting infections at their new destinations. They may spread the disease from one country to another depending on their premorbid state, length of stay, and mode of transportation [3–5]. Some examples of diseases reported to have been related to travel include Ebola, Zika, YF, Cholera, and, more recently, COVID-19 [6–9].

The WHO recommends mandatory vaccination against YF to stop the YF virus from being imported into vulnerable countries [10]. In addition, the YF vaccine is recommended for people aged nine months or older and traveling to or living in areas at risk for YF virus in Africa and South America [11]. Despite this guidance, imported cases of YF have been documented in non-endemic countries due to a lack of vaccinations for travelers [8]. Similarly, there is a risk of Cholera infection in travelers to areas where Cholera is prevalent [12]. One hundred fifty cases of travel-related Cholera infections have been documented in different countries between 1990 and 2018 [13]. In Nigeria, between weeks 1 and 39 of 2020 [14], 1140 suspected Cholera cases, 40 laboratory-confirmed confirmed cases, and 63 deaths were reported compared with 14,310 suspected Cholera cases with 389 laboratory-confirmed and 236 deaths during the same period in 2018 [15]. Besides the associated morbidity and mortality, food-borne conditions impede socioeconomic development by straining healthcare systems, harming national economies, tourism, and trade [16]. Plague often manifests as a severe illness [17]. An outbreak of Plague occurred in Madagascar in 2017, following which the WHO identified a probable case of Plague in a traveler who had returned from Madagascar to Seychelles [18, 19].

To prepare for, detect, and respond to YF epidemics, the Nigerian Government rolled out a national guideline for YF preparedness and response in 2019 [20]. This guideline aligns with the global Eliminate yellow fever YF Yellow Fever epidemics (EYE) strategy 2017–2026 guideline. In addition, the Nigeria Centers for Disease Control (NCDC) does weekly epidemiological reporting of epidemiological-prone diseases.

To mitigate the risk of person-to-person transmission of infectious diseases, prophylactic health measures are crucial for both travelers and the general population [21]. Some examples of prophylactic measures include pre-travel vaccination, booster vaccination, serology monitoring, and other preventive measures such as taking precautions against flea and insect bites, of using prophylactic medications, avoiding direct contact with infected body fluids and tissues, and not handling animal carcasses [22–24]. Pre-travel health assessments or consultation for travelers is carried out to promote risk reduction through preventive measures, including ensuring that travelers seek health advice before embarking on international travel and are up-to-date with their immunizations [25]. International travelers are expected to seek medical consultation at least 4 to 8 weeks before starting their journey [26].

There is lack of data on pre-travel health practices against Cholera, YF, and Plague, a group of infectious diseases endemic in Africa and subject to the IHR of 1969 and 2015. Previous studies have assessed vaccination and pre-travel health practices against YF among travelers from non-endemic areas (such as the USA and Europe) to endemic areas in Africa [27–29]. Still, there is needs to be more research that assesses the vaccination practices of travelers from developing countries to other parts of the world. Also, to our knowledge, there has been no airport-based study on pre-travel health practices against a group of diseases subject to IHR. Therefore, we aimed to generate and describe the baseline travelers’ pre-travel health practices towards Cholera, YF, and Plague at Murtala Muhammed International Airport (MMIA) in Nigeria.

## Methods

### Study design

A cross-sectional study design was employed to collect data from travelers embarking on international travel through the airport.

### Study setting

This study was conducted at Murtala Muhammed International Airport (MMIA) in Nigeria. Nigeria’s premier international air gateway. MMIA is located in Lagos and was commissioned in 1978 [30]. According to the Nigerian Bureau of Statistics, 3,202,837 passengers traveled through the international airport in 2019 [31]. The Port Health Services, a government agency located within the airport arena, provides pre-travel health vaccination services. The activities at the airport are overseen by the Federal Airport Authority of Nigeria (FAAN).

### Recruitment process

This study was planned for January 2020, but data collection was carried out from October to November 2020 after easing the COVID-19 lockdown in Nigeria. Data collection took place daily at the departure gates of MMIA based on a pre-determined and Institutional Review Boards (IRB) approved study protocol.

### Participants – inclusion and exclusion criteria and sample size

We included travelers aged 18 years and above and excluded travelers using MMIA for transfer or transit to other countries. A brief screening survey was conducted to identify eligible participants. The sample size was determined using Leslie Fisher’s formula for prevalence studies. The assumed proportion of YF vaccination was 76% from a previous study, and the level of precision was set at 0.04 [32]. The computed sample size was increased by 10% to compensate for non-responses. This gave a total sample size of 486.

### Participants recruitment: sampling technique and selection of study participants

#### Stage 1: selection of travelers’ destinations based on WHO regions

Four out of the six WHO regions were included in this study [33]. The four areas were African, American, European, and Eastern Mediterranean because Nigeria does not have direct flights to the remaining two regions (i.e. South-East Asian and Western Pacific Regions).

#### Stage 2: proportional allocation of travelers to WHO regions using a stratified sampling method

Based on the 2019 Winter flight schedule received from the Commercial Travel Section of the Operation Unit of MMIA, the expected monthly traveler volume was estimated to be 223,704. This was proportionally allocated to 4 WHO regions to ensure the representativeness of travelers and travel destinations. The African region, comprising 113,316, was further stratified into the West African, East African, Central African, and Southern African regions.

#### Stage 3: selection of study participants using simple random sampling

A simple random sampling method was used to select study participants per region. The samples were randomly selected using electronically generated random numbers. The data collection was completed in four weeks using a daily data collection plan to meet the data target for each WHO region. Questionnaires were administered at the departure gates of each flight. If a selected traveler declines to participate in the study, the traveler representing the following randomly selected number was approached to participate.

### Data collection

A semi-structured questionnaire, adapted from the International Health Travel Questionnaires [7, 13] and questionnaires from previous studies, was used to collect relevant data from study participants (supplementary document 1). The questionnaire was pre-tested at the Nnamdi Azikiwe International Airport, Abuja, Nigeria.

### Variables

The **independent** variables of interest were educational level, nationality, Cholera endemicity at the destination, YF endemicity at the destination, traveler’s awareness of pre-travel vaccination, and inspection of vaccination cards during the previous trip(s). Their covariates are listed as a footnote under Table 1.

**Table 1:** Multivariable logistic regression analysis of the factors associated with pre-travel health practices among respondents

Variables	n (%)	COR (95% Confidence limit)	p-value	AOR^a^ (95% Confidence limit)	p-value
Educational Level^b^					
Secondary/high school or less *(Ref)*	85 (17.7)				
Undergraduate or College	201 (42.0)	2.31 (0.92, 5.77)	0.07	2.91 (1.10, 7.70)	0.03
Graduate (Masters/Ph.D./Professional)	193 (40.3)	1.11 (0.42, 2.97)	0.84	1.89 (0.62, 5.77)	0.26
**Nationality (Nigerian vs. Non-Nigerian)** ^**c**^					
Non-Nigerian *(Ref)*	158 (33.0)				
Nigerian	321 (67.0)	0.27 (0.15, 0.50)	< 0.01	0.53 (0.18, 1.55)	0.25
**Endemicity of Cholera infection at destination** ^**d**^					
No *(Ref)*	378 (78.9)				
Yes	101 (21.1)	3.35 (1.83, 6.14)	< 0.01	1.35 (0.41, 4.48)	0.62
**Endemicity of YF infection at destination** ^**d**^					
No *(Ref)*	429 (89.6)				
Yes	50 (10.4)	0.71 (0.24, 2.05)	0.52	3.31 (1.41, 7.77)	0.01
**Awareness of pre-travel vaccinations** ^**e**^					
Yes (Ref)	217 (45.3)				
No	262 (54.7)	1.96 (1.05, 3.64)	0.03	1.32 (0.63, 2.76)	0.46
**Inspection of vaccination card during the previous travel** ^**f**^					
No *(Ref)*	117 (24.4)				
Yes	362 (75.6)	1.58 (0.74, 3.34)	0.24	0.79 (0.33, 1.86)	0.58

*(Ref) Reference Variable. COR: Crude Odds Ratio*

^a^AOR (95% CI) = Adjusted odds ratio and 95% confidence interval for multivariable logistic regression with adjustment for covariates

^b^Educational, level was adjusted for age, gender, marital status, occupation, monthly income, and religion

^c^Nationality was adjusted for the class of country of residence, Area of dwelling at the destination, and WHO region of residence

^d^Endemicity of Cholera and YF infections were adjusted for the perceived risk of contracting the diseases at the destination, educational status, proposed length of stay at the destination, the purpose of traveling, and the knowledge of Cholera, YF, and Plague, respectively

^e^Pre-travel vaccination was adjusted for educational status, age, nationality, religion, pre-travel consultation, and awareness of cholera and plague

^f^Inspection of the vaccination card during previous travel was adjusted for nationality, Class of Country of residence, Class of country traveled to during previous journey, trip travel without being vaccinated, and the knowledge of Cholera, YF, and Plague respectively

The **dependent** variable is pre-travel health practices (a composite score of the practice of pre-travel consultation/advice, pre-travel vaccination status, and pre-travel preventive measures). Pre-travel consultation or advice was defined as information obtained from a health professional [22]. Pre-travel consultation was categorized as taken and not taken; vaccination was categorized as vaccinated and not vaccinated based on reported vaccination for YF among those traveling to endemic countries. Self-reported YF vaccination was used as a proxy for vaccination because it was readily accessible at the Nigerian Port Health Services compared to the Cholera vaccine. To validate the vaccination status, we inspected the vaccination cards of travelers. Pre-travel preventive measures were categorized as taken and not taken among travelers going to YF endemic and Plague endemic regions. The preventive measures assessed were actions to prevent (a) the bite of infected vector fleas for Plague and mosquitoes for YF, (b) contact with infectious bodily fluids or contaminated materials, and (c) the inhalation of respiratory droplets/small particles from a patient with pneumonic Plague.

The composite score of pre-travel health practices ranged from 0 to 3. Having taken pre-travel consultation/advice was scored 1 while not taken was scored 0, pre-travel vaccination was scored 1 while non-vaccination was scored 0, and having taken pre-travel preventive measures was scored 1 while non-practice was scored 0. Those with a score of 3 were categorized as having good practices, while those with scores of 2 or less were categorized as having poor practices. We ensured no overlap of the scores to prevent misclassification of travelers.

### Statistical analysis

Data analysis was done using IBM SPSS Statistics 25.0. Descriptive statistics were presented in frequency tables, while pre-travel health practices were visually represented using a pie chart. A binary logistic regression analysis was employed to examine the association between the independent and dependent variables of interest and pre-travel health practices. Furthermore, a multivariable analysis was conducted to reduce the influence of potentially confounding variables, controlling for relevant covariates (Table 1). Before fitting the model, variables with cell counts less than 5 were recategorized. Educational level was recategorized into 3 levels: secondary/high school or less, undergraduate or College, and Graduate (Masters/Ph.D./Professional). The undergraduate or College and Graduate (Masters/Ph.D./Professional) were also referred to as higher educational levels under the discussion.

We used a directed acyclic graph (DAG) to identify possible confounders of our selected exposures and controlled for them in the multivariable analysis. The significance level was set at 5%, and the odds ratio was presented with a 95% confidence interval.

## Results

Four hundred and seventy-nine valid questionnaires were analyzed. The median age of respondents was 34.0 years (IQR = 28.0, 44.0). As regards the gender of respondents, 332 (69.3%) were males, giving an M:F ratio of 2.3 to 1. One hundred and eighty-five (38.6%) were single, and 201 (42.0%) had an undergraduate degree (Table 2). Twenty-six airlines from 21 countries were operating at MMIA as of the time of this study. The top three nationalities of the travelers who participated in this study were Nigeria 321 (67.0%), India 91 (19.0%), and Ghana 20 (4.1%). In contrast, the top three places our respondents were traveling to were the UAE 99 (20.7%), India 88 (18.3%), and the United Kingdom 61 (12.7%), respectively. Using the World Economic Situations and Prospects (WESP) [34] classification, travelers were mostly from developing countries 451 (94.2%) (Table 2).

**Table 2:** Demographic characteristics of respondents and respondents’ destinations

Variable	Frequency (n = 479)	Percentage (%)
**Age Groups**		
18–29	159	33.2
30–39	153	31.9
40–49	99	20.7
50 and above	68	14.2
**Gender**		
Female	147	30.7
Male	332	69.3
**Marital Status**		
Single	185	38.6
Married	275	57.4
Divorced/Separated	5	1.0
Widowed/widower	14	2.9
**Educational Status**		
No formal	1	0.2
Primary	19	4.0
Secondary	65	13.6
Undergraduate	201	42.0
Postgraduate	193	40.3
**Occupation**		
Unemployed (Student/trainee/Retired)	89	18.6
Employed	390	81.4
**Monthly income in Naira**		
Less than or equal to 30,000	33	6.9
30,001 to 120,000	164	34.2
120,001 to 240,000	154	32.2
240,001 to 480,000	76	15.9
480,001 to 960,000	32	6.7
Greater than 960,000	20	4.2
**Religion**		
Christianity (Orthodox, Protestant, Catholic)	333	69.5
Islam	42	8.8
Others (Buddhism, Hindu, Sikhism, Paganism)	98	20.5
None	6	1.3
**Nationality**		
Nigerian	321	67.0
Non-Nigerian	158	33.0
**WHO Region of Residence**		
AFRO	350	73.1
SEARO	91	19.0
AMRO	16	3.3
EURO	15	3.1
EMRO	6	1.3
WPRO	1	0.2
**Class of Country of Residence** ^**#**^		
Developing	451	94.2
Developed	28	5.8
**Region respondents are traveling to**		
EURO	135	28.2
EMRO	109	22.8
SEARO	92	19.2
AMRO	78	16.3
AFRO	63	13.2
WPRO	2	0.4
**Area of dwelling at destination**		
Urban	476	99.4
Rural	3	0.6
**Endemicity of diseases subject to IHR at travelers’ destination**		
No	328	68.5
Yes		
Cholera	101	21.1
YF	50	10.4
Plague	0	0.0

*Multiple responses were allowed

AFRO: African Region EMRO: Eastern Mediterranean Region EURO: European Region

AMRO: American Region SEARO: Southeast Asia Region WPRO: Western Pacific Region

^*#*^*Country of residence was classified into developed and developing according to the country classification system of the 2020 World Economic Situations and Prospects (WESP)* [41].

**Fig. 1:**
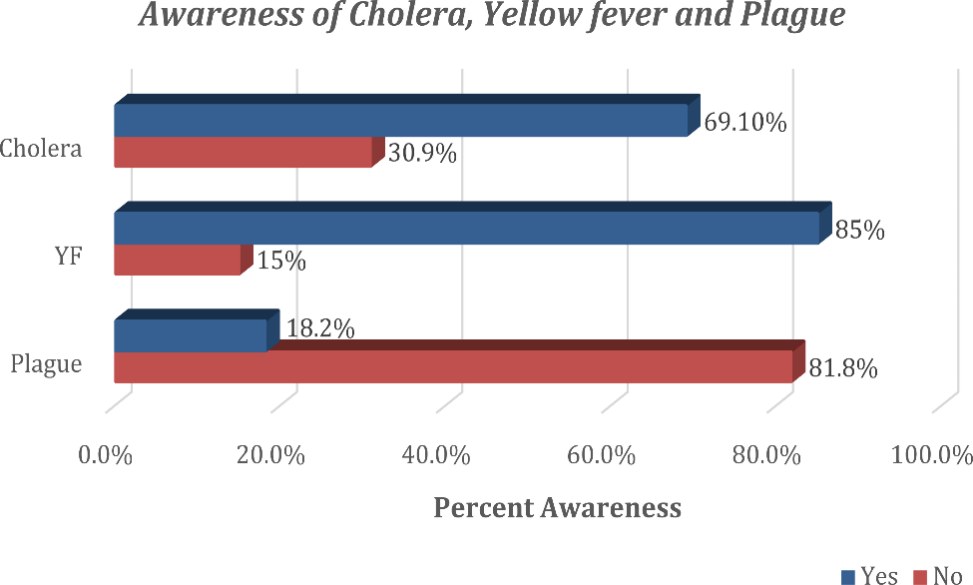
Awareness about Cholera, YF and Plague among study participants

Overall, 59 (12.3%) were not aware of any of the three diseases, while 90 (18.8%) were aware of only one of the diseases, 255 (53.2%) were aware of two conditions, and 75 (15.7%) were of all the three diseases. Their sources of information about these diseases included friends/relatives 31 (6.5%), social media/internet 61 (12.7%), and health professionals 27 (5.6%), amongst others. In terms of travelers’ awareness of the specific diseases, 331 (69.1%) were aware of Cholera, 407 (85.0%) were aware of YF, and 87 (18.2%) travelers were aware of the Plague (Fig. 1). Of the 87 travelers aware of Plague disease, 2 (0.4%) could identify avoiding dead animals as a way of protecting themselves from acquiring the infection. Also displayed in Table 2 is the Endemicity of Cholera, YF, or Plague diseases at travelers’ final destination. One hundred and one (21.1%) of participants were traveling to Cholera-endemic countries, and 50 (10.4%) were traveling to YF-endemic countries.

As displayed in Tables 3, 311 (64.3%) of the total respondents were aware of pre-travel consultation. Their sources of information, amongst others, include friends/relatives in 180 (37.6%) travelers, social media/internet in 155 (32.4%) travelers, and health professionals in 102 (21.3%) travelers. Two hundred and seventy-one (56.6%) of travelers who were aware of pre-travel consultation have ever done it. The time interval between receiving pre-travel consultation and departure time was less than one week in 227 (47.4%) travelers (Table 3). Two hundred and sixty-two (54.7%) were aware of pre-travel vaccination, 156 (32.6%) reported to have been vaccinated before embarking on this journey, 260 (54.3%) said to have traveled in the past without being vaccinated, and another 120 (25.1%) had traveled before without holding vaccination cards. During this study, 105 (21.9%) traveled without holding vaccination cards. The self-reported vaccination rate for YF and Cholera were 29.4% and 1.9%, respectively; however, among all travelers, only 271 (56.6%) provided vaccination cards for inspection as evidence of claim. Travelers’ practices of preventive measures against insect bites are also displayed in Table 3. Overall, 226 (47.2%) respondents were ready to protect themselves against insect bites.

**Table 3:** Awareness and practice of travelers on pre-travel consultation, pre-travel vaccination among respondents, and preventive measures against diseases subject to IHR

Variable	Frequency	Percentage (%)
**Awareness about pre-travel consultation (n = 479)**		
No	168	35.7
Yes	311	64.3
Source of Information* (n = 311)		
Friends/relatives	180	37.6
Social media/internet	155	32.4
Health professional/travel medicine specialist	102	21.3
Embassy	37	7.7
Travel agent/Others	27	5.6
Print media/Mass media	26	5.4
Employer	16	3.3
**Pre-travel consultation/health advice taken (n = 311)**		
Not taken	40	8.4
Taken	271	56.6
**Reason for taking pre-travel consultation (n = 271)**		
It is the right thing to do	198	41.3
For pre-travel vaccinations	40	8.4
My employer recommended it	18	3.8
Due to my known health condition	15	3.1
**Type of clinic where pre-travel consultation was taken (n = 271)**		
Specialist Hospital (Non-travel medicine specialist)	168	35.1
General health clinics (General Practitioner)	40	8.4
Travel Clinic (Travel medicine specialist)	34	7.1
Others (e.g., Company clinic)	19	4
Port Health Service /Aviation Medical Clinic	10	2.1
**Time to travel when pre-travel consultation was taken (n = 271)**		
Less than 1 week	227	47.4
1 to 2 weeks	31	6.5
2 to 4 weeks	2	0.4
4 to 8 weeks	4	0.8
More than 8 weeks	7	1.5
**The reason why pre-travel consultation was not sought or taken (n = 40)**		
I did not find it important	35	7.3
There was no time to do so before my trip/busy	2	0.4
The difficulty of geographical access	1	0.2
I did not know about it	1	0.2
Others	1	0.2
**Awareness about pre-travel vaccination (n = 479)**		
No	217	45.3
Yes	262	54.7
**Previous travel-related vaccination (n = 262)**		
Not vaccinated	106	22.1
Vaccinated	156	32.6
**Reason for taking pre-travel vaccination (n = 156)**		
To safeguard my health	109	22.8
It is the right thing to do	35	7.3
As part of the requirements for a visa	9	1.9
My employer recommended it	3	0.6
Reason for not taking pre-travel vaccination (n = 106)*		
I did not find it important	78	16.3
I refused it	9	1.9
I am not aware of it	9	1.9
To avoid side effects of vaccination	5	1
There was no time to do so/busy	4	0.8
Financial reason/cost was high	1	0.2
**Previous travel without being vaccinated (n = 479)**		
No	219	45.7
Yes	260	54.3
**Previous travel without holding a vaccination card (n = 479)**		
No	359	74.9
Yes	120	25.1
Travel Vaccines taken*		
YeF	141	29.4
Cholera	9	1.9
**Ways by which travelers have prepared to protect themselves against insect bites during**		
stay at destination (n = 479)*		
Use of insect repellants	222	46.3
Use of insecticide-treated nets	79	16.5
Use of air conditioner	51	10.6
Clothes that can cover arms and legs	27	5.6
Use of insecticide sprays	7	1.5
I will avoid staying out at night	1	0.2

*Multiple responses allowed

WHO does not recommend vaccination against plague for travelers; therefore, plague vaccination was not captured in this study.^17^

**Fig. 2:**
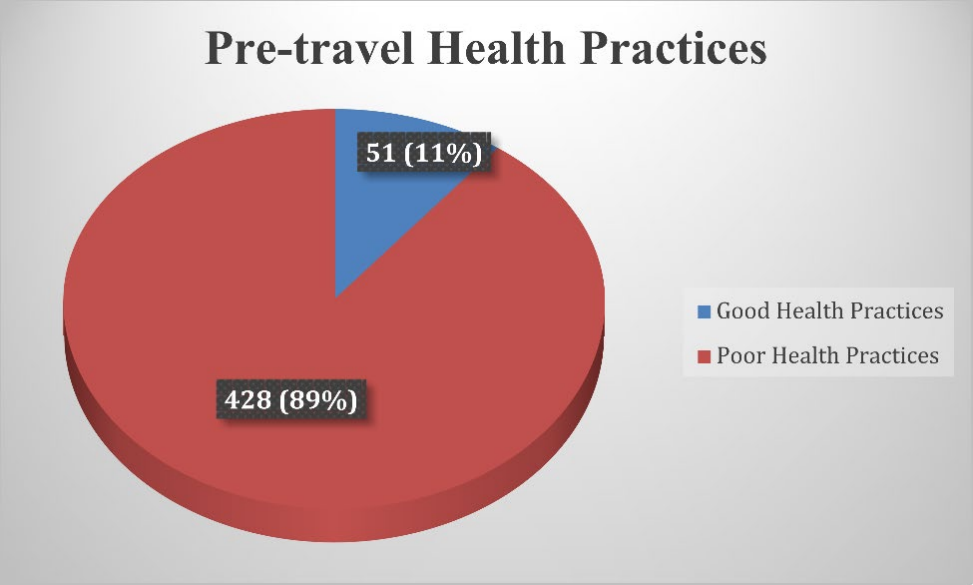
Status of pre-travel health practices among study participants

Figure 2 shows the status of pre-travel health practices among respondents. Of the 479 travelers, 51 (10.6%) had good practices, while 428 (89.4%) had poor practices.

Travelers with bachelor/college degrees, when compared to those with secondary/high school education, had 2.91 times higher odds of having good practices when adjusting for other factors (95% C.I: 1.10, 7.70; p < 0.03). Also, those traveling to destinations endemic for YF infection, when compared to those who are not traveling to endemic countries/areas, had 48% lower odds of having good practices when adjusting for other factors (95% C.I: 1.41, 7.77; p < 0.01) (Table 1).

## Discussion

Our study fills a significant research gap and was the first to investigate travelers’ pre-travel health practices towards Cholera, YF, and Plague at MMIA in Nigeria.

The male-to-female ratio found in our study can be explained by the fact that there are more males than females in Nigeria [35].

Our study found social media/websites to be one of the primary sources of information on the awareness of the diseases subjected to IHR. In contrast, a previous study at MMIA reported that 77.4% of travelers mentioned travel agents as their primary source of information [36].

The difference could be attributed to the focus of their study, which was on general information for travelers rather than on disease-specific information. Health professionals were also found to rank among the first three leading sources of information for travelers on awareness of YF, awareness of Plague understanding, and pre-travel health consultation or advice. These two primary sources (social media/internet and health professionals) may serve as channels for disseminating information about pre-travel health preparedness for international travelers.

In this study, only one-tenth of travelers had good pre-travel health practices against the diseases subject to IHR. This rate is below expectation as we expected at least 31.1% (i.e., the proportion of participants traveling to countries endemic for Cholera, YF, and Plague) to have good pre-travel health practices. The implication is that many travelers could risk being infected with Cholera, YF, or Plague at their destinations. Only a few (2.3%) of those seeking pre-travel consultation did so more than four weeks before their journey. This low proportion is far from expectation when evaluated against the recommendation of WHO that consultation should be done at least 4–8 weeks before a proposed journey [5]. The public health implication of seeking consultation late or not taking pre-travel consultation is that travelers may not get adequate information about the endemic diseases at their proposed destinations or may not have enough time to take preventive measures, including vaccinations.

Notably, despite the YF outbreak in Nigeria at the time of this study, only one-third of travelers took the YF vaccine [37]. This proportion was lower than the vaccination rate in previous studies conducted at MMIA and Ethiopia, where 66.2% and 83.1% of participants reported being vaccinated, respectively [38, 39]. The low rates of Plague awareness and practice of preventive measures against insect bites are not unexpected as Plague is not a common disease globally and is not endemic in Nigeria.

Having good health practices among travelers to countries endemic to YF could be partially attributed to historical knowledge of the disease among people living in Nigeria and widespread access to the internet by international travelers. The relationship between higher educational levels and pre-travel health practices has also been established in a study conducted in Taiwan [40]. This association could be attributed to high curiosity for knowledge among travelers with advanced and widespread access to the internet.

### Research limitation

This study left out two WHO regions (South-East Asian and Western Pacific Regions) in selecting study participants. However, those regions were considered and included in categorizing and analyzing travelers’ final destinations.

### Research strength

This study was the first to examine a combination of diseases subject to international health regulation. In addition, our analysis used a directed acyclic graph (DAG) to identify and control for possible confounders of our selected exposures.

### Generalizability

This study can be generalized because of its large sample size and the stratified selection of study participants to ensure the representativeness of participants. We also used a multivariate statistical method to identify the predictors of good pre-travel health practices.

## Conclusion

This study revealed that good pre-travel health practices against diseases subject to IHR were deficient among international travelers departing from MMIA. Travelers’ educational status and travel destinations were predictors of good practices.

One crucial recommendation to the Nigerian Government is to include travelers’ health in the existing secondary/high school curriculum on health. This initiative will build a culture of harnessing travel-related health information among students as they grow. We envisage this would have a ripple effect on travelers and the general population. There is a need for the Government of Nigeria to implement public enlightenment programs on pre-travel health consultation and pre-travel vaccination; we believe that social media will be a vital tool in achieving this by the result of this study. Healthcare practitioners are also encouraged to give their clients adequate information about pre-travel health preparations when they visit for pre-travel health consultations.

### Electronic supplementary material

Below is the link to the electronic supplementary material.

Supplementary Material 1

## Data Availability

The datasets used and analyzed during the current study are available from the first author upon reasonable request.

## Abbreviations

AFRO: African Regional Office
AMRO: American Regional Office
AOR: Adjusted Odds Ratio
COR: Crude Odds Ratio
EMRO: Eastern Mediterranean Regional Office
EURO: European Regional Office
IHR: International Health Regulations
IRB: Institutional Review Boards
IQR: Interquartile Range
SEARO: Southeast Asia Regional Office
SPSS: Statistical Package for the Social Sciences
UAE: United Arab Emirates
WESP: World Economic Situations and Prospects
WHO: World Health Organization
WPRO: Western Pacific Regional Office
YF: Yellow Fever
