# Supplementary material for: International health regulations and pre-travel health practices of international travelers at Nigerian airport: a cross-sectional study
Source: Trop Dis Travel Med Vaccines. 2023 Dec 5;9:21. doi: 10.1186/s40794-023-00207-8 (PMC10696719; doi:10.1186/s40794-023-00207-8)
Supplement: Supplementary file 1 — Supplementary Material 1 [file 40794_2023_207_MOESM1_ESM.pdf]

International health regulations and pre-travel practices of international travellers at Nigerian airport: a cross-sectional study

QUESTIONNAIRE

IDENTIFICATION

1) Questionnaire serial number ___________________

2) Date of interview ____________________________

3) Location _________________________________

S/No	Question	Response	Remark
**I**	**SOCIODEMOGRAPHIC CHARACTERISTICS**		
101	Age as at last birthday (in years)	______________________________	
102	Gender	A) Male<br>B) Female<br>C) I would rather not disclose	
103	Marital status	A) Single<br>B) Married<br>C) Divorced/separated<br>D) Widow/widower<br>E) Others<br>Please Specify_____________	
104	Educational status<br>*(Highest certificate obtained)*	A) No formal<br>B) Primary/Elementary/Grade 6<br>C) Secondary/High school/Grade 12<br>D) Undergraduate/College<br>E) Postgraduate (Masters/PhD/Fellowship)	
105	Occupation	______________________________	

106	Monthly income (in US dollars)		
107	Religion	A) Christianity<br>B) Islam<br>C) Others<br>Please specify_______________	

II	TRAVEL PATTERN		
201	Nationality?	____________________<br><br>Country	
202	Which area of the country do you live?	A) Urban (≥5,000 population)<br>B) Rural (<5,000 population)	Continue on question 203
203	Were you treated for any infection before embarking on this journey?	A) Yes<br>B) No	If yes, continue on question 204<br><br>If no, skip to question 205
204	If yes, which one?<br><br>(Please write Diagnosis in the next column)		Continue on question 205
205	Which country are you travelling to (final destination)?<br><br>(Not lay over country)	____________________<br><br>Country	
206	Which area of that country will you stay?	A) Urban (≥5,000 population)<br>B) Rural (<5,000 population)	Continue on question 207
207	How long will you be there?	A) Less than 24 hours<br>B) 1 to 7 days<br>C) 1 to 4 weeks<br>D) 1 to 6 months<br>E) Longer than 6 months	Continue on question 208
208	What is your purpose of travelling?	A) Study<br>B) Work<br>C) Volunteer/humanitarian<br>D) Visiting friends or relatives/ Tourism/vacation<br>E) Returning home<br>F) Conference/ Official meetings<br>G) Accompanying relatives/officials<br>H) Others<br>Please specify_______________	Continue on question 209

209	Has your international certificate of vaccination and prophylaxis ever been inspected?	A) Yes<br>B) No	If yes, continue on question 210<br><br>If no, skip to question 301
210	If yes, which country (ies)?	____________________<br><br>Country	Continue on question 301
**III**	**AWARENESS ABOUT CHOLERA, YELLOW FEVER AND PLAGUE**		
301	Have you heard of Cholera before?	A) Yes<br>B) No	If yes to question 301, continue on question 302<br><br>If No, skip to 306
302	What was your source of information?<br><br>(Multiple responses allowed)	A) Friends/ relatives<br>B) Social media/internet<br>C) Print media/mass media<br>D) Embassy as part of Visa Application<br>E) Employer<br>F) Health professionals/Travel clinic<br>G) Travel agent/ travel book<br>H) Others<br>Please specify__________	Continue on question 303
303	Is Cholera a problem in the country you are travelling to?	A) Yes<br>B) No<br>C) Don't know/Not sure	If yes, continue on question 304<br><br>If no, skip to 306
304	How can travellers protect themselves from Cholera?<br><br>(Multiple responses allowed)	A) Use of safe food and water<br>B) Good personal hygiene<br>C) Proper environmental sanitation<br>D) I've been vaccinated<br>E) Use of insecticide treated nets<br>F) Use of insect repellents<br>G) Avoid dead animals remains and tissues<br>H) I do not know of any	Continue on question 305

305	Have you heard about Cholera vaccine before?	A) Yes<br>B) No	If yes to question 305, continue on question 306<br><br>If no, skip to 306
	**ABOUT YELLOW FEVER**		
306	Have you heard of Yellow fever before?	A) Yes<br>B) No	If yes, continue on question 307<br><br>If no, skip to 311
307	What was your source of information?<br><br>(Multiple responses allowed)	A) Friends/ relatives<br>B) Social media/internet<br>C) Print media/mass media<br>D) Embassy as part of Visa Application<br>E) Employer<br>F) Health professionals/Travel clinic<br>G) Travel agent/ travel book<br>H) Others<br>Please specify__________	Continue on question 308
308	Is Yellow fever a problem in your country of destination?	A) Yes<br>B) No<br>C) I don't know	If yes, continue on question 309<br><br>No/ don't know, skip to 311
309	How can travellers protect themselves from yellow fever?<br><br>(Multiple responses allowed)	A) Taking clean and safe food<br>B) Good personal hygiene<br>C) Proper environmental sanitation<br>D) Use of insecticide treated nets<br>E) Use of insect repellents<br>F) Avoid dead animals remains and tissues<br>G) I've been vaccinated<br>H) I don't know	Continue on question 310
310	Have you heard about Yellow fever vaccine?	A) Yes<br>B) No	If yes, continue on question 311<br><br>If no, skip to 311

	**ABOUT PLAGUE**		
311	Have you heard of Plague before?	A) Yes<br>B) No	If yes, continue on question 312<br><br>If no, skip to 401
312	What was your source of information?<br><br>(Multiple responses allowed)	A) Friends/ relatives<br>B) Social media/internet<br>C) Print media/mass media<br>D) Embassy as part of Visa Application<br>E) Employer<br>F) Health professionals/Travel clinic<br>G) Travel agent/ travel book<br>H) Others<br>Please specify___________	Continue on 313
313	Is plague a problem in your country of destination?	A) Yes<br>B) No<br>C) I don't know	If yes, continue on question 314<br><br>If no, skip to 401
314	How can travellers protect themselves from plague?<br><br>(Multiple responses allowed)	A) Taking clean and safe food<br>B) Good personal hygiene<br>C) Use of insecticide treated nets<br>D) Use of insect repellents<br>E) Avoid dead animals remains and tissues<br>F) Vaccination<br>G) I do not know of any	Continue on question 315
**IV**	**PRE-TRAVEL HEALTH CONSULTATION STATUS**		
401	Have you heard about pre-travel health advice/consultation before?	A) Yes<br>B) No	If yes, continue on question 402<br><br>If no, skip to question 501
402	What is the source of your information?	A) Friends/ relatives<br>B) Social media/internet<br>C) Print media/mass media	Continue on question 403

	(Multiple responses allowed)	D) Embassy as part of Visa Application<br>E) Employer<br>F) Health professionals/Travel clinic<br>G) Travel agent/ travel book<br>H) Others<br>Please specify___________	
403	Have you ever taken pre-travel health consultation?	A) Yes<br>B) No	If yes, continue on question 404<br><br>If No, skip to question 405
404	If yes, why?	A) Because it is the right thing to do<br>B) Because my employer recommended it<br>C) Because of requirement for pre-travel vaccination<br>D) Because of my known health condition<br>E) Others<br>Please specify_____________	Continue on question 406
405	If No, why not?	A) Because of financial reasons (cost is high)<br>B) Because I did not find it important<br>C) Difficulty of geographical access<br>D) There was no time to do so before my trip<br>E) I did not know about it<br>F) Others<br>Please specify_____________	Continue on question 501
406	Where did you take your pre-travel advice?	A) Port Health Service / Aviation Medical Clinic<br>B) Travel clinic (Travel medicine specialist)<br>C) General health centre/hospital (from General practitioners)<br>D) Specialist hospital (Non-travel medicine specialist)<br>E) Others<br>Please specify_____________	Continue on question 407
407	How long before travel did you seek pre-travel health advice?	A) Less than 1 week<br>B) 1 to 2 weeks	Continue on question 501

<table>
<tr><td></td><td></td><td>C) 2 to 4 weeks
D) 4 to 8 weeks
E) More than 8 weeks</td><td></td></tr>
<tr><td></td><td colspan="3"></td></tr>
<tr><td>V</td><td colspan="3">VACCINATION HISTORY AND STATUS; AND PREVENTION MEASURES</td></tr>
<tr><td>501</td><td>Have you heard of pre-travel vaccination before?</td><td>A) Yes
B) No</td><td>If yes, continue on question 502

If no, skip to question 507</td></tr>
<tr><td>502</td><td>Have you travelled out of Nigeria in the past without being vaccinated?</td><td>A) Yes
B) No</td><td>Continue on question 503</td></tr>
<tr><td>503</td><td>Have you ever taken any pre-travel vaccinations before?</td><td>A) Yes
B) No</td><td>If yes, continue on question 504

If no, skip to question 506</td></tr>
<tr><td>504</td><td>If yes, which vaccines have you taken?

At the end of this questionnaire, you will be asked to show your vaccination certificate</td><td>Please mark all vaccinations taken below

E.g. Hepatitis A (√)

<table>
<tr><th>S/No</th><th>Vaccine type</th><th>Mark (√)</th></tr>
<tr><td>1</td><td>Hepatitis A</td><td></td></tr>
<tr><td>2</td><td>Hepatitis B</td><td></td></tr>
<tr><td>3</td><td>Typhoid</td><td></td></tr>
<tr><td>4</td><td>Cholera</td><td></td></tr>
<tr><td>5</td><td>Yellow fever</td><td></td></tr>
<tr><td>6</td><td>Rabies</td><td></td></tr>
<tr><td>7</td><td>Meningitis</td><td></td></tr>
<tr><td>8</td><td>Measles Mumps Rubella</td><td></td></tr>
</table></td><td>Continue on question 505</td></tr>
</table>

<table>
<tr><td></td><td></td><td><table><tr><td>9</td><td>Tetanus, Diphtheria & Pertusis (DPT)</td><td></td></tr><tr><td>10</td><td>Chicken pox</td><td></td></tr><tr><td>11</td><td>Shingles</td><td></td></tr><tr><td>12</td><td>Pneumonia</td><td></td></tr><tr><td>13</td><td>Influenza</td><td></td></tr><tr><td>14</td><td>Polio</td><td></td></tr></table></td><td></td></tr>
<tr><td>505</td><td>If yes to question 503, why?</td><td>A) Because it is the right thing to do
B) Because my employer recommended it
C) Because of requirement for pre-travel vaccination
D) To protect my health
E) Others
Please specify_____________</td><td>Continue on question 507</td></tr>
<tr><td>506</td><td>If No to question 503, why not?</td><td>A) Because of financial reasons (cost is high)
B) Because I did not find it important
C) Difficulty of geographical access
D) There was no time to do so before my trip
E) I did not know about it
F) To avoid side effects
G) I refused it
H) Required vaccine(s) not available
I) Not taken because of pregnancy
J) Others
Please specify_____________</td><td>Continue on question 507</td></tr>
<tr><td>507</td><td>In what ways have you prepared to protect yourselves against insect bites during your stay at your final destination?

(Multiple responses allowed)</td><td>A) Use of insecticide sprays
B) Use of insect repellents
C) Clothes that can cover arms and legs
D) Use of Insecticide treated nets
E) Use of air conditioners
F) I will not go out at night
G) Others
Please specify_____________</td><td>Continue on question 508</td></tr>
</table>

508	Will you like to participate in future research on this topic?	A) Yes<br>B) No	If yes, continue on question 509<br><br>If No, skip to 510
509	If yes, can you please share your email?<br><br>(Please write your email address in the next column)		Continue on question 510
510	Can you show us your international certificate of vaccination?	A) Yes<br>B) No<br>C) I do not have<br>D) It is not right here with me	***For Interviewer use only***<br><br>Shown____<br><br>Not shown____
511	Did COVID-19 influence your health preparations for this trip?	A) Yes<br>B) No	If yes, continue on question 512
512	If yes, in what ways?	A) I sought health advice / took health consultation<br>B) I decided to take necessary precautions including use of PPEs<br>C) I took trial covid-19 vaccine<br>D) I took other relevant pre-travel vaccines<br>E) I had covid-19 testing<br>F) Others ___________________	

Thank you very much for your time, we wish you a safe journey.
